# Intermittent Fasting and Its Effects on Weight, Glycemia, Lipids, and Blood Pressure: A Narrative Review

**DOI:** 10.3390/nu15163661

**Published:** 2023-08-21

**Authors:** Elie Naous, Angela Achkar, Joanna Mitri

**Affiliations:** 1 Division of Internal Medicine, St. Elizabeth’s Medical Center, Tufts University School of Medicine and Boston University School of Medicine, Brighton, MA 02135, USA; angela.achkar@steward.org; 2 Joslin Diabetes Center, Harvard Medical School, Boston, MA 02215, USA; joanna.mitri@joslin.harvard.edu

**Keywords:** intermittent fasting, body mass index, glycemia, lipid profile, blood pressure

## Abstract

Metabolic syndrome (MetS) has become a significant public health concern globally. Weight managementis crucial in controlling MetS risk factors, making energy balance and weight loss strategies important in nutrition recommendations. Intermittent fasting (IF) has gained traction as a dietary approach for weight management and cardiovascular risk reduction. However, the effects of IF on cardiovascular risk factors have been inconsistent in previous studies. This review aims to summarize the effects of various types of IF on body mass index (BMI), glycemia, lipid profile, and blood pressure, while providing insights into their clinical implications. A comprehensive search of interventional studies and meta-analyses was conducted, and the results were analyzed. The findings indicate that different types of IF lead to mixed effects. Time-restricted eating (TRE) and alternate-day fasting (ADF) consistently showed decreases in BMI, while the outcomes of intermittent energy restriction (IER) were more uncertain. The effects of IF on glycemia and lipid profile were also variable, with TRE and ADF generally showing positive results. However, the impact of IER remained inconsistent. More research is needed to understand the long-term effects and optimal implementation of IF for managing metabolic syndrome and cardiovascular risk factors.

## 1. Introduction

Metabolic syndrome (MetS) is defined by the American Heart Association (AHA) as the presence of three or more of the following risk factors: insulin resistance (IR) indicated by fasting glucose levels >100 mg/dL, hypertension > 135/85 mmHg, hypertriglyceridemia > 150 mg/dL, reduced high-density lipoprotein cholesterol (HDL-C) levels (<40 mg/dL for men and <50 mg/dL for women), and elevated waist circumference (>102 cm for men and >88 cm for women) [1]. In the 21st century, MetS has emerged as a primary risk factor for atherosclerotic cardiovascular disease (ASCVD) [2]. The prevalence of these metabolic risk factors has reached pandemic levels globally over the past five decades, making it a significant public health concern [3,4,5]. In the United States, diabetes, hypertensive diseases, and ischemic heart diseases have consistently ranked among the top ten leading causes of death and disability over the past two decades [6]. Weight management has been shown to play a crucial role in controlling these risk factors [7], making energy balance and weight loss strategies important considerations in nutrition recommendations [8]. Various lifestyle modifications, including physical activity, psychological interventions to address unhealthy behaviors, and dietary interventions aiming at promoting weight loss and preventing weight regain have been employed in the treatment of obesity [9]. Several dietary patterns, such as the Dietary Approaches to Stop Hypertension (DASH) and Mediterranean diets, have demonstrated beneficial effects on metabolic markers, including improvements in lipid profile and blood pressure (BP) [10,11]. Additionally, the Nordic diet, characterized by high consumption of mushrooms, fish, fruits, and vegetables, can improve lipid profile [12]. Plant-based diets, such as the vegan diet, have also shown protective effects against MetS [2].

Recently, emerging evidence has suggested that the timing of meals, in addition to the content of the diet, may influence the progression of chronic diseases [13]. As a result, intermittent fasting (IF) has gained traction as a dietary approach for weight management [14], diabetes [15], dyslipidemia [16], and cardiovascular risk reduction [14]. However, the findings from meta-analyses and randomized clinical trials (RCTs) investigating the effects of IF on cardiovascular risk factors have been conflicting. While some studies have shown positive outcomes in terms of weight loss [17,18], others have reported no significant advantage over continuous calorie restriction [19]. Similarly, the effects of IF on glycemia have yielded mixed results, with some studies indicating improvements [20] and others showing no significant differences [21]. The impact of IF on lipid profiles has also been variable, with the effectiveness of different IF patterns remaining uncertain [22].

This review aims to summarize the effects of various types of IF on body mass index (BMI), glycemia, lipid profile, and blood pressure, while providing insights into their clinical implications.

## 2. Approaches to IF

IF encompasses various eating habits, and different types of IF have been proposed and implemented [23]. Table 1 provides a definition of the IF diets included in the cited studies.

## 3. Methods

We conducted a comprehensive search for interventional studies (Table 2) and meta-analyses of cohort studies (Table 3) that investigated the effects of IF on body weight (BW), body fat (BF), BMI, glycemia, lipid profile, and BP. Systematic reviews conducted without meta-analyses were excluded from the analysis. Our search was performed using the PubMed database and included articles published from January 2010 to January 2023. We employed specific medical subject heading (MeSH) terms, such as intermittent fasting AND type OR glucose OR diabetes OR lipid profile OR cardiovascular diseases OR blood pressure AND randomized controlled trial OR meta-analyses. Relevant articles were carefully reviewed, and data related to the intervention and type of IF, study duration, number of participants, and effects on BW, BMI, glycemia, lipid profile, and blood pressure were extracted. We excluded articles pertaining to religious fasting, such as Ramadan fasting and Orthodox fasting.

## 4. Results

### 4.1. Effects of IF on BW, BF, and BMI:

We identified a total of 22 interventional studies (Table 2) that examined the impact of IF on BW, BF, and BMI. The study populations ranged from 10 to 150 overweight or obese individuals, and the duration of the studies varied from 1 to 60 weeks. Among these studies, 14 reported a reduction in BW compared to the baseline after implementing IF [20,21,22,24,25,26,27,28,29,30,31,32,33,34]. Eight studies indicated a greater weight loss in the IF group compared to the control groups [24,26,27,35,36,37,38,39], while another eight studies found no significant difference in weight loss between the IF and control groups [19,22,25,28,31,33,34,40]. TRE and ADF consistently showed decreases in BW, BF, and BMI, whereas the outcomes of IER were more uncertain. When no control group was included, and groups were followed over time, TRE demonstrated beneficial effects on reducing BW in both obese and overweight individuals compared to their baseline [20,21,39]. However, no subgroups based on BMI categories were studied. Several RCTs utilized control groups with no intervention on diet and found that CCR, ADF, and TRE were effective in reducing BW and BMI in the overall population [24,32,36]. It is important to note that the study conducted by Moro et al. [36] included a group of 34 resistance-trained men. This particular inclusion of participants with prior training experience may introduce a confounding bias to the interpretation of the results obtained from the diet interventions. Additionally, Varady et al. [37], in their RCT, demonstrated that ADF significantly reduced BW and BF compared to a no-intervention group in individuals with normal BMI. This suggests that IF has positive effects not only on overweight or obese individuals but also on those with a BMI in the normal range. When comparing different types of IF to CER/CCR, the results were divergent. Two RCTs indicated a reduction in BMI in both CER and ADF groups with no significant difference in weight loss between them [19,40]. However, these two studies included healthy overweight/obese individuals and excluded those with cardiovascular risk factors, limiting the generalizability of the results to unhealthy patients. Six RCTs reported a reduction in BMI in CER and IER groups, but no significant difference was observed between the two groups [22,25,28,31,33,34]. This beneficial effect was observed in both obese and overweight patients, but no statistical analysis based on BMI categories was performed. Notably, half of these RCTs [22,33,34] exclusively included patients with diabetes, and Sundfor et al. [28] included individuals with at least one comorbidity. These findings suggest that IER may not have a superiority over CER in unhealthy populations. Only two RCTs demonstrated the superiority of IER over CER in weight reduction [26,38]. However, the study conducted by Byrn et al. [26] exclusively included sedentary male individuals at baseline, limiting the generalizability of the findings. A noteworthy RCT [30] conducted on a population of 101 overweight and obese patients reported a greater effect of ADF compared to TRE (*p* < 0.001) in BW reduction after a 3-week follow-up. However, caution should be exercised when interpreting these results due to the very short follow-up period. Overall, few RCTs considered behavioral lifestyle modifications during the study period. Additionally, it is important to acknowledge the potential impact of the Hawthorne effect and observer bias in the studies where daily meal records were based on nutritionist interviews and self-reported food logs.

Concerning the eight meta-analyses discussing the effect of IF on BW and included in this review (Table 3), the number of integrated studies ranged from 6 to 43. Three studies [17,44,45] showed a reduction of BMI and BW in the IF groups in comparison to baseline, whereas five other studies [14,15,18,46,47] showed a reduction in comparison to control groups. Moreover, one of these meta-analyses [46] concluded that there was no difference between IF and calorie restriction groups on weight loss. Notably, the latter mixed different types of IF, which can affect its reliability and interpretability. Moreover, the long-term effects of IF subgroups were not exhaustively analyzed in detail due to the limited number of studies, weakening the statistical significance of the results.

### 4.2. Effects of IF on Glycemia

We identified 18 interventional studies (Table 2) that investigated the effects of IF on fasting glucose (FG), insulin levels, and HbA1c. The number of overweight or obese participants ranged from 8 to 137, and the study durations varied from 4 days to 60 weeks. These studies yielded mixed results. Eight studies [19,20,22,27,34,38,41,42] reported improved glycemia in the IF groups compared to the baseline. Moreover, four studies [25,27,32,43] demonstrated a positive effect of IF on blood glucose levels compared to groups with CER or no-intervention diets. However, seven studies [19,22,30,34,35,38,40] found no significant difference in glycemic parameters between the IF groups and control groups, whether it was a no-intervention diet group or CER. Three studies [21,29,33] reported that IF had no effect on blood glucose and insulin. Surprisingly, one study [31] even indicated an increase in FG in the IF group. Most of the studies showed a positive effect of TRE and ADF on glycemia, whereas the effect of IER remained inconsistent. When individuals on a TRE diet were followed over time without a control group, Wilkinson et al. [20] demonstrated a decrease in FG and insulin levels, whereas Gabel et al. [21] found no effect on FG and insulin levels. In comparison to a no-intervention diet, ADF was associated with improved FG. This finding was supported by Buthani et al. [32] in their RCT, which included 64 obese individuals followed over 3 months. When comparing ADF to CER/CCR, no significant difference was found between these two groups in terms of glucose metabolism in the two RCTs [19,40]. However, it is worth noting that these studies only included healthy individuals without a baseline of diabetes or prediabetes. Therefore, the advantage of ADF over CER in patients with pre-diabetes/diabetes cannot be conclusively determined. The results were contradictory when comparing IER to CER/CCR. Carter et al. conducted two RCTs [22,34] that showed a reduction in HbA1c in both groups compared to baseline, but no superiority was found between the two groups. Interestingly, in their second RCT [34], including 92 patients with diabetes followed over 116 weeks, the improvement in HbA1c in both groups was more significant when the baseline HbA1c was above 8% (*p* < 0.001). Harvie et al. [25] demonstrated an improvement in fasting insulin levels in the IER group compared to the CER group in a population of 107 healthy women with a BMI >25 kg/m^2^. Therefore, the beneficial effect of IER over CER was limited to healthy women and could not be extrapolated to unhealthy women or men. Additionally, in an RCT including healthy individuals at baseline, fasting glucose showed a worsening trend in the IER group compared to the CER group (*p* = 0.008) [31]. However, this study was underpowered, with only 27 participants, so the results should be interpreted cautiously.

Furthermore, six meta-analyses (Table 3) provided conflicting results. The number of integrated studies ranged from 6 to 43. Two meta-analyses showed a reduction in FG levels in the IF groups [17,45], while another study found no significant difference between IF and CER groups in terms of blood glucose levels [14]. Similarly, Borgundvaac et al. [15] found no significant difference between IF and no-intervention groups in terms of HbA1c reduction. TRE was shown to better reduce FG levels compared to no-intervention groups [48]. Finally, Gu et al. [46] described no difference between IF and CR or no-intervention groups in terms of blood glucose levels, but they found a reduction in fasting insulin levels and insulin resistance compared to control groups with no intervention on diet. It is worth noting that the meta-analysis showing a significant reduction in FG levels included twice the number of studies compared to those that did not show significant effects. Consequently, these small-sized meta-analyses lack sensitivity. Moreover, Cho et al. [17] only included healthy individuals, which limits the generalizability of the results to patients suffering from metabolic diseases who could potentially benefit from these diets.

### 4.3. Effects of IF on Lipid Profile

Fifteen interventional studies (Table 2) were conducted to investigate the effects of IF on lipid profiles. The study participants ranged from 8 to 137 individuals, and the duration of the studies varied from 4 days to 116 weeks. Among the three types of IF considered, TRE demonstrated the most favorable outcomes on lipid profiles across the majority of the studies.

However, it is important to note that the studies examining the effects of IF on lipid profiles varied significantly in terms of study design, parameters assessed, number of participants, and baseline population characteristics. These differences introduced potential biases, including selection bias, confounding variables, and observer bias, which could have influenced the study outcomes.

Studies by McAllister et al. [29] and Jamshed et al. [43] reported that TRE, whether isocaloric or ad libitum, resulted in increased levels of HDL-C (*p* = 0.005 and *p* = 0.03, respectively). Conversely, Gabel et al. [35] and Sutton et al. [41] did not observe any significant differences in low-density lipoprotein cholesterol (LDL-C) and HDL-C levels (*p* = 0.54, *p* = 0.75 on LDL-C; *p* = 0.11, *p* = 0.48 on HDL-C, respectively) when compared to matched control groups. Kahleova et al. [27] showed a reduction in LDL-C levels compared to baseline, with no significant differences observed when compared to a six-meals-daily-based diet (*p* = 0.82 for LDL-C and *p* = 0.3 for TG).

Regarding ADF, Bhutani et al. [32] found no significant effects on LDL-C (*p* = 0.90), HDL-C (*p* = 0.80), and TG (*p* = 0.30). Similarly, when compared to a CCR group, ADF did not significantly influence LDL-C (*p* = 0.40) and HDL-C (*p* = 0.99) [19]. However, Trepanowski et al. [40] demonstrated that a prolonged ADF diet resulted in increased LDL-C levels compared to a CCR diet, while TG levels did not differ significantly between the two groups.

IER showed some effects on lipid profile. Harvie et al. [25] and Kunduraci et al. [38] reported improvements in LDL-C and TG levels compared to baseline, with no significant differences observed when compared to a CER group. Consequently, no superiority of IER over the usual CER was demonstrated. Similarly, Carter et al. [33] found no differences in LDL-C and TG levels between IER and CER groups, but unexpectedly, they observed a decrease in HDL-C levels compared to baseline in the IER group (*p* = 0.02).

This review also included six meta-analyses (Table 3) that investigated the effects of IF on lipid profiles. The number of studies included in these meta-analyses ranged from 6 to 43. Three of the meta-analyses [14,15,48] concluded that there were no significant differences between IF and CER or no-intervention groups. However, Meng et al. [16] noted a decrease in total cholesterol and LDL-C levels in the IF group, with no significant effect on TG levels. On the contrary, two other studies [45,46] showed a reduction in TG levels in the IF groups. These meta-analyses differed in terms of daily calorie intake, fasting days per week, intervention duration, and timing and duration of the fasting window, which contributed to the discordant results. Moreover, the timing of lipid panel measurements, particularly for TG, was notably different among the studies, as TG levels tend to fluctuate throughout the day.

### 4.4. Effects of IF on BP

Ten interventional studies [20,21,25,31,32,35,37,38,40,41] were conducted to examine the effects of IF on systolic blood pressure (SBP) and diastolic blood pressure (DBP) (Table 2). IF, in all its studied types, showed a significant reduction in both SBP and DBP compared to baseline. This finding is consistent with the well-established correlation between weight loss, which was observed in most of the cited studies, and BP control.

TRE was found to have a positive influence on BP, as several studies [20,21,35,41] reported significant reductions in both SBP and DBP compared to baseline (*p* = 0.041, *p* < 0.05, *p* = 0.02, and *p* = 0.03, respectively).

ADF was also shown to reduce SBP and DBP compared to a no-intervention diet in two different studies [32,37] (*p* < 0.05 for SBP and DBP, *p* = 0.02 for SBP, and *p* = 0.03 for DBP, respectively).

BP levels decreased when following the IER diet. Harvie et al. [25] and Kunduraci et al. [38] observed improvements in both SBP and DBP compared to baseline, with no significant differences found when comparing each group to the regular CER diet. These findings suggest that the reduction in blood pressure is likely attributed to weight loss rather than intermittent fasting (IF) itself. On the other hand, Antoni et al. [31] found a greater reduction in SBP in the IER group compared to the CER group over a 60-week period (*p* = 0.02). Therefore, definitive conclusions regarding the benefits of IER versus CER in relation to blood pressure reduction cannot be reached at this time.

Furthermore, four meta-analyses (Table 3) were included in this review to study the effects of IF on BP. The number of studies included in these meta-analyses ranged from 6 to 43. Except for Moon et al. [45], who showed a reduction in BP in the IF groups, all other studies [14,15,46] concluded that there were no significant differences between IF and CER or no-intervention groups regarding BP. It is worth mentioning that a considerable portion of the meta-analyses that showed no effect of IF on BP included studies lacking randomization. Additionally, the timing of BP measurements and dietary sodium intake were not standardized in most of the studies, contributing to the heterogeneity of the results. Furthermore, the impact of BP on arterial remodeling often requires several years to become clinically relevant and the studies included in the analysis did not incorporate long-term follow-up periods. Assessing arterial remodeling over an extended duration would provide a more comprehensive understanding of its clinical significance.

## 5. Discussion

Based on the data collected, it appears that IF has positive effects on cardiovascular (CV) risk factors. IF has been shown to lead to weight loss, improvements in glucose metabolism, lipid profile (reducing LDL-C and increasing HDL-C), and control of systolic and diastolic blood pressure compared to baseline. IF achieves these effects by reducing visceral and truncal fats through energy deficits, which improves the leptin/adiponectin ratio and appetite control [49]. Additionally, the reduction in adiposity and chronic inflammation contributes to a decrease in insulin resistance [50]. In terms of lipid regulation, IF reduces hepatic production of very low-density lipoprotein cholesterol (VLDL-C) and TG production while increasing fatty acid oxidation. IF is also associated with increased expression of peroxisome proliferator-activated receptor-α (PPAR-α) and peroxisome proliferator-activated receptor γ (PPAR-γ) coactivator 1 α in the liver, leading to increased fatty acid oxidation and production of Apo A, as well as decreased synthesis of Apo B, ultimately contributing to the reduction of LDL-C and VLDL-C [51]. The effects of IF on blood pressure are mediated by the activation of the parasympathetic system in the brainstem [52].

Paradoxically, when compared to other more regular types of diets, particularly CCR/CER, IF does not appear to be superior in terms of BMI, diabetes, and lipid profile in some of the studies included in this review. These findings could be attributed to various factors.

Genetics play a role in how different individuals respond to various diets. Certain genetic variations can affect the efficacy of a diet for weight maintenance, as demonstrated by studies on the A-allele of the FTO gene variant rs9939609, which showed that its presence in obese patients complicates long-term weight maintenance after initial weight loss following a low-calorie diet [53]. Conducting more genetic studies to understand the genes and alleles responsible for the variability of responses to IF in different populations would be beneficial.

Furthermore, despite the lack of specific recommendations regarding the types of food to be consumed during IF diets, the quantities of sugar, saturated fat, cholesterol, and sodium were not significantly different from more conventional weight loss diets [54]. In addition, understanding the physiology of IF, such as the activation of lipolysis and ketogenesis [55], as well as the improvement of appetite control through increased peptide YY [56], helps clarify how successful weight loss can be achieved. Numerous studies have shown that weight loss improves blood pressure [57], glucose levels [58], and lipid profile [59], thus supporting the findings of this review.

Moreover, emerging evidence suggests that the higher the adherence to any type of diet, the greater the success rate [60]. IF allows flexibility in food choices during the eating window [54], which presumably improves compliance. Compliance with IF has been investigated in multiple studies [25,31,61] and has shown a range of adherence rates from 43% to 93%. This lack of food intake restrictions facilitates adherence to this type of diet, resulting in better control of CV risk factors.

Finally, different signaling pathways and circulating ketones during fasting periods can enhance overall health and cellular resistance to disease, even in cases where IF does not lead to weight loss [62]. This highlights the superiority of IF over more traditional diets in terms of cellular metabolism, even when both types of diets are equally effective in improving CV risk factors.

## 6. Clinical Approaches and Reflections

In our review, most studies have shown a positive effect of IF on the control of BMI, glucose metabolism, lipid profile (increase in HDL-C and reduction in LDL-C), and blood pressure. Similarly, Malinowski et al. [52] demonstrated in their overview that the IF diet contributes to the control of many risk factors, such as diabetes, lipid profile, blood pressure, and obesity, which is related to protection against the development of ASCVD. However, Allaf et al. [63] demonstrated in their systematic review that although IF was superior to ad libitum eating in reducing weight, there was no significant clinical difference between IF and CER in improving cardiometabolic risk factors, and hence, no reduction in ASCVD. Consequently, further research is needed to understand the long-term clinical outcomes of IF on ASCVD independently from its effects on cardiovascular risk factors. Moreover, it has been shown that ADF leads to a significant reduction in high-sensitivity C-reactive protein (hsCRP). Considering that patients with elevated hsCRP are at high risk for ASCVD events, IF can be an effective approach to preventing ASCVD in patients with elevated hsCRP levels. It would be interesting to identify patients who may benefit the most from this type of diet.

While the short-term effects of IF have been found to be beneficial in some studies, the long-term effects remain uncertain. These patterns of calorie restriction carry the risk of potential metabolic rate reduction in the long term, hindering the maintenance or pursuit of weight loss, in agreement with the fasting ancestral theory [64]. Therefore, further prospective studies should be conducted to follow the outcomes of IF over several years.

Another important clinical outcome is related to the IF’s psychological and psychiatric influence on individuals. A recent study [65] demonstrated that fasting participants are at a higher risk of binge eating compared to individuals who have never fasted (*p* = 0.03). This supports the research suggesting that IF can be linked to a higher risk of disordered eating behaviors, making it unfavorable for patients with a history of eating disorders. Additionally, although this review highlights the benefits of IF on metabolic health, the implementation of these dietary interventions can be challenging in the modern lifestyle, particularly in social settings with different mealtimes [66]. This factor can affect social life and increase the risk of depression. Individuals should, therefore, be encouraged to choose the fasting approach that can be most easily incorporated into their lifestyle.

It is worth mentioning that glycemic fluctuations appear to be the most significant concern during IF. It has been shown that IF increases the risk of hypo- and hyperglycemia in patients with diabetes [67]. Consequently, IF should not be recommended for people at risk of glycemic fluctuations, such as pregnant women, patients with uncontrolled diabetes, and the elderly, due to the increased risk of falls and fractures [68].

On a different note, emerging research has demonstrated that IF can reduce androgen production in both men and women, therefore leading to potential negative effects on libido. However, intriguingly, this side effect holds promising implications for women suffering from polycystic ovary syndrome [69].

Finally, IF appears to exert an effect on bone homeostasis, as evidenced by its association with an elevated risk of osteoporosis, rendering it an unfavorable dietary choice among the elderly [70].

## 7. Strengths and Limitations

There are potential limitations to the available data that are worth mentioning. First, the studies included in this review exhibit heterogeneity in their methods, emphasizing the importance of combining and comparing their results. Second, various factors such as genetics, physical activity, and social habits may need to be consideredwhen analyzing results; however, most of these studies were randomized controlled trials, reducing this bias. Finally, there is a limited number of interventional studies, and most of them have small sample sizes with limited follow-up durations. For instance, no long-term support was provided to evaluate patients’ compliance and long-term effects. However, our review constitutes an extensive assessment of IF and its effects on most cardiovascular risk factors by providing an overview and summary of different types of studies.

## 8. Conclusions

In conclusion, while IF appears to show promise as a dietary approach for weight management and improving metabolic markers associated with MetS, some uncertainties cast doubt on its efficacy, safety, and long-term effects. Consequently, it is essential to be cautious about drawing definitive conclusions on its long-term impact on health, given the current gaps in the design of the available studies. Healthcare professionals should consider individualized approaches and closely monitor patients undergoing IF, taking into account their specific health conditions and goals. 

## Figures and Tables

**Table 1:** Types of IF included in this review.

Types of Fasting	Characteristics
Alternate-day fasting (ADF)	24-h severe calorie restriction (25% restriction below estimated requirements) every other day.
Time-restricted eating (TRE)	Restriction of food intake < 10 h/day and fasting period > 14 h/day.
Intermittent energy/calorie restriction (IER/ICR)	Severe calory restriction (25% restriction below estimated requirements) on some days of the week and ad libitum intake on the rest.
Twice-per-week fasting (TWF) *	Subtype of IER/ICR: severe calory restriction (25% restriction below estimated requirements) on 2 days of the week and ad libitum intake on the 5 other days.

* Twice-per-week fasting (TWF), also known as the 5:2 diet, is a widely recognized dietary approach. For consistency throughout this study, we will use the term TWF (generic name) to refer to the 5:2 diet (trade name).

**Table 2:** Summary of interventional studies investigating effects of IF on BW, BF, BMI, glycemia, BP, and lipid profile.

Author (Year)	N, Patients	Duration (Weeks)	Design	Group 1	Group 2	Effect on Weight, Fat, and BMI	Effect on Glycemia	Effects on Lipid Profile	Effects on Blood Pressure
Catenacci et al. (2016) [19]	26 obese	8	RCT	ADF	CCR	No significant difference in weight loss between groups (*p* = 0.45)	↓ Fasting glucose in ADF (*p* = 0.01) with no difference between groups (*p* = 0.55)	↓ Total cholesterol, LDL-C and HDL-C in both groups with no difference between groups (*p* = 0.3, *p* = 0.4, *p* = 0.99 respectively)	-
Wilkinson et al. (2020) [20]	19 overweight or obese	12	Single-arm, paired-sample trial	TRE (10-h time-restricted eating)	No control	↓ BW (*p* = 0.00028) in comparison to baseline	↓ Fasting glucose (*p* = 0.08), and insulin (*p* = 0.06) in comparison to baseline. No significant difference for HbA1c (*p* = 0.058)	↓ LDL-C (*p* = 0.016) in comparison to baseline	↓ SBP (*p* = 0.041 and DBP (*p* = 0.004) in comparison to baseline
Gabel et al. (2020) [21]	14 obese	12	Prospective study	TRE (8-h time-restricted eating)	No control	↓ BW (*p* < 0.05) in comparison to baseline	No effect on fasting glucose and insulin	-	↓ SBP (*p* < 0.05) in comparison to baseline
Carter et al. (2016) [22]	63 overweight or obese	12	RCT	IER	CER	↓ BW (*p* < 0.001) with no difference between groups (*p* = 0.7)	↓ HbA1c (*p* < 0.001) with no difference between groups (*p* = 0.3)	-	-
Schübel et al. (2018) [24]	150 overweight or obese	12	RCT	ICR or CCR	No intervention on diet	↓ BW ICR > CCR > control (*p* < 0.001)	-	-	-
Harvie et al. (2011) [25]	107 overweight or obese	24	RCT	IER	CER	↓ BW and fat mass in comparison to baseline without differences between groups (*p* = 0.26)	↓ insulin fasting levels and insulin resistance in comparison to CER (*p* = 0.04)	↓ LDL-C and triglycerides in comparison to baseline with no differences between groups (*p* = 0.93 and 0.64, respectively)	↓ SBP and DBP in comparison to baseline with no differences between groups (*p* = 0.99 and 0.84, respectively)
Byrne et al. (2018) [26]	47 obese	16	RCT	IER	CER	↓ BW in both groups with greater loss in IER (*p* < 0.01)	-	-	-
Kahleova et al. (2014) [27]	54 overweight or obese	12	Cross over RCT	TRE (8-h time-restricted eating)	Diet of 6 meals a day	↓ BW and BMI (*p* < 0.001) in both groups, more in TRE	↓ Fasting glucose (*p* < 0.001) in both groups, more in TRE	↓ LDL-C and TG in both groups, no difference between groups (*p* = 0.82 for LDL-C and *p* = 0.3 for TG)	-
Sundfor et al. (2018) [28]	112 overweight or obese	24	RCT	IER	CER	↓ BW with no difference between groups (*p* = 0.6)	-	-	-
McAllister et al. (2019) [29]	22 overweight or obese	4	RCT	Isocaloric TRE	Ad libitum TRE	↓ BW in both groups (*p* < 0.001) in comparison to baseline	No effect on blood glucose levels (*p* = 0.26)	↑ HDL-C in both groups (*p* = 0.005) No effect on LDL-C (*p* = 0.64)	-
Chair et al. (2022) [30]	101 overweight or obese	3	RCT	ADF	TRE (8-h time-restricted eating)	↓ BW in both groups with ↓ ADF> TRE (*p* < 0.001)	No difference between groups on ↓ fasting glucose	↑ HDL-C in both groups with ↑ ADF > TRE (*p* < 0.009) No difference between groups on ↓ LDL-C ↓ Triglycerides TRE > ADF (*p* = 0.01)	-
Antoni et al. (2018) [31]	27 overweight or obese	60	RCT	TWF	CER	↓ BW in both groups with no difference between groups (*p* = 0.43)	↑ fasting glucose in IER group (*p* = 0.008)	-	↓ SBP IER > CER (*p* = 0.02) and ↓ DBP without differences between groups (*p* = 0.7)
Bhutani et al. (2013) [32]	64 obese	12	RCT	ADF	No-intervention diet	↓ BW and BMI in the ADF group (*p* < 0.05) ↓ Fat mass (*p* < 0.01)	↓ Fasting blood glucose in ADF in comparison to the control group (*p* < 0.05)	No effect on LDL-C (*p* = 0.9), HDL-C (*p* = 0.8), and TG (*p* = 0.3)	↓ SBP and DBP (*p* < 0.05)
Carter et al. (2019) [33]	137 normal weight or overweight or obese	48	RCT	IER	CER	↓ BW with maintenance in both groups (*p* < 0.001) with no differences between groups (*p* = 0.19)	No effect on HbA1c and fasting glucose level (*p* = 0.14, *p* = 0.46, respectively) for both groups in comparison to baseline.	No differences for LDL-C and TG in both groups (*p* = 0.02), ↓ HDL-C in both groups (*p* = 0.02)	-
Carter et al. (2018) [34]	97 overweight or obese	116	RCT	TWF	CER	↓ BW with no difference between groups (*p* = 0.25)	↓ HbA1c (*p* < 0.001) with no difference between groups (*p* = 0.65) Higher change when HbA1c baseline >8% (*p* < 0.001)	↓ LDL-C, TG, ↑ HDL-C (*p* < 0.01) with no difference between groups	-
Gabel et al. (2018) [35]	23 obese	12	Prospective study	TRE (8-h time-restricted eating)	Matched historical control group	↓ BW and BMI in comparison to control (*p* < 0.001)	No statistical difference (*p* = 0.77) for fasting glucose and (*p* = 0.16) for fasting insulin in comparison to control group	No statistical difference on lipid profile (*p* = 0.54 for LDL-C and *p* = 0.11 for HDL-C)	↓ SBP (*p* = 0.02) and DBP (*p* = 0.41)
Moro et al. (2016) [36]	34 overweight	8	RCT	TRE (8-h time-restricted eating)	No intervention on diet	↓ Fat mass in comparison to control (*p* = 0.0448)	-	-	-
Varady et al. (2013) [37]	32 normal weight or overweight	12	RCT	ADF	No intervention on diet	↓ BW and fat mass in comparison to control (*p* < 0.001)	-	↓ LDL-C (*p* = 0.01) in comparison to baseline with no statistical difference between groups	↓ SBP (*p* = 0.02) and DBP (*p* = 0.03) levels in comparison to baseline with no statistical difference between groups
Kunduraci et al. (2020) [38]	70 overweight or obese	12	RCT	IER	CER	↓ BW IER > CER (*p* < 0.003)	↓ HbA1c in both groups (*p* < 0.001) with no difference between both groups (*p* = 0.777)	↓ LDL-C and TG in both groups with no difference between groups (*p* = 0.76, 0.36, respectively)	↓ SBP and DBP in both groups with no difference between groups (*p* = 0.15, 0.28 respectively)
Bhutani et al. (2010) [39]	16 obese	10	Single-arm Interventional Human Study	ADF	2 weeks baseline control (usual eating and exercise habits)	↓ BW, BMI, and fat mass (*p* < 0.05) in comparison to control	-	↓ LDL-C and TG (*p* < 0.05) in comparison to control No effect on HDL-C	-
Trepanowski et al. (2017) [40]	100 overweight or obese	48	RCT	ADF	CCR	No significant difference in weight loss between groups	No significant difference in fasting glucose and insulin	No significant difference in lipid profile	No significant difference in BP
Sutton et al. (2018) [41]	8 obese	5	Cross over RCT	TRE (6-h time-restricted eating)	Control (12-h eating period)	-	↓ fasting insulin in comparison to baseline (*p* = 0.05)	No effects on LDL-C (*p* = 0.75) and HDL-C (*p* = 0.48)	↓ SBP and DBP in comparison to control (*p* = 0.03)
Hutchison et al. (2019) [42]	15 obese	1	Cross over RCT	TRE early	TRE late	-	↓glucose levels and fasting insulin (*p* < 0.001) with no effect of mealtime (*p* > 0.66)	-	-
Jamshed et al. (2019) [43]	11 overweight and obese	0.6	Cross over RCT	TRE (6-h time-restricted eating)	Control (12-h eating period)	-	↓ 24-h mean glucose levels in comparison to control (*p* = 0.0003) ↓ fasting glucose (*p* = 0.02) and insulin (*p* < 0.0001) in comparison to control	↑ HDL-C (*p* = 0.03) and LDL-C (*p* = 0.02) in the morning but no effect in the evening in comparison to control group	-

Key: ↓ decrease; ↑ increase; - not studied. IF = intermittent fasting; RCT = randomized controlled trial; ICR = intermittent calorie restriction, CCR = continuous calorie restriction, IER = intermittent energy restriction; CER = continuous energy restriction; TRE = time-restricted eating; ADF = alternate-day fasting; BW = body weight; BF = body fat; BMI = body mass index; SBP = systolic blood pressure; DBP = diastolic blood pressure; TG = triglycerides; LDL-C = low-density lipoprotein cholesterol; HDL-C = high-density lipoprotein cholesterol.

**Table 3:** Summary of meta-analyses investigating effects of IF on BW, BF, BMI, glycemia, lipid profile, and BP.

Author (Year)	Number of Included Studies	Effect on Body Weight, Body Fat, and BMI	Effect on Glycemia	Effect on Lipid Profile	Effect on Blood Pressure
Harris et al. (2018) [14]	6	↓ BW similar between IF and CER (WMD: −1.03 kg; 95% CI −2.46 kg to 0.40 kg; *p* = 0.156). ↓ BW IF > no intervention (WMD: 4.14 kg; 95% CI 6.30 kg to 1.99 kg; *p* < 0.001).	Improvement of insulin concentration in IF in comparison to CER (WMD: −4.66 pmol/L −9.12 pmol/L to −0.19 pmol/L; *p* = 0.041). No significant difference between IF and CER on glucose.	No significant difference between IF and CER on LDL-C, HDL-C, and TG.	No significant difference between IF and CER.
Borgundvaac et al. (2021) [15]	7	↓ BW IF> no intervention 1.89 kg (95% CI, −2.91 to −0.86 kg).	No significant difference between IF and no-intervention groups on HbA1c reduction –0.11% (95% CI, −0.38% to 0.17%).	No significant difference between IF and no-intervention groups on LDL-C, HDL-C, and TG.	No significant difference between IF and no-intervention groups.
Meng et al. (2020) [16]	28	-	-	↓ TC (WMD −2.99 mg/dL, 95% CI −8.6 to 2.62) with IF. ↓ LDL-C (WMD −5.48 mg/dL, 95% CI −8.71 to −2.24) with IF. No significant effect on TG (95% CI −13.07 to 1.7) with IF.	-
Cho et al. (2019) [17]	12	↓ BMI 0.75 kg/m2 (95% CI, −1.44 to −0.06; *p* = 0.033) in IF groups. ↓ BW 1.94 kg (95% CI, −5.20 to 1.31; *p* = 0.241) in IF groups.	↓ Fasting glucose (−4.16 mg/dL; 95% CI, −6.92 to −1.40; *p* = 0.003) in IF groups.	-	-
Guerrero et al. (2021) [18]	18	↓ BW IF > no intervention.	-	-	-
Patikorn et al. (2021) [44]	11	↓ BW with TWF diet (95% CI −2.79 to −0.55).	-	-	
Moon et al. [45]	19	↓ BW in TRE group (−0.90; 95% CI: −1.71 to −0.10), ↓ BF in TRE group (−1.58, 95% CI: −2.64 to −0.51).	↓ FG in TRE group (−2.96; 95% CI, −5.60 to −0.33).	↓ TG in TRE group (−11.60, 95% CI: −23.30 to −0.27).	↓ SBP in TRE group (−3.07; 95% CI: −5.76 to −0.37).
Gu et al. (2022) [46]	43	↓ BW and BMI in IF in comparison to no-intervention group (WMD = 1.10, 95% CI: 0.09–2.12 and WMD = 0.38, 95% CI: 0.08–0.68, respectively). No difference between IF and CR in weight loss.	No difference between IF and no-intervention group on fasting glucose (95% CI: (−0.14) − 0.13, *p*= 0.94). ↓ Fasting insulin levels and ↓ insulin resistance in IF in comparison to the no-intervention group (95% CI: 0.02–0.40, *p* = 0.03 and CI: 0.04–0.65, *p* = 0.03, respectively). No difference between IF and CR on glucose levels.	↓ TG in IF in comparison to no-intervention group (95% CI: 0.09–0.35, *p* = 0.001). No difference between IF and no-intervention groups on LDL-C and HDL-C (*p* = 0.42 and *p* = 0.63 respectively). No difference between IF and CR in blood lipids.	No effect on SBP and DBP (WMD = 1.32, *p* = 0.33, and WMD = 0.96, *p* = 0.39).
Ashtary-Larky et al. (2021) [47]	8	↓ BW (WMD = −2.08 kg; 95% CI: −3.04, −1.13), BF (WMD = −1.36 kg; 95% CI: −1.94, −0.78), and BMI (WMD = −0.52 kg/m^2^; 95% CI: −0.85, −0.19) in IF in comparison to no-intervention group.	-	-	-
Maranhao Pureza et al. (2021) [48]	18	-	↓ fasting glucose TRE > no-intervention diets (WMD −2.75; 95% CI [−4.59; −0.90] mg/dL; *p* < 0.01). No difference in insulin levels between TRE and no-intervention groups (WMD −1.54; 95% CI [−3.35; 0.26] mUI/L; *p* = 0.09).	No difference in TG (1.61; 95% CI [−7.82; 11.05] mg/dL, *p* = 0.73), total cholesterol (6.33; 95% CI [−3.45; 16.12] mg/dL, *p* = 0.20), LDL-C (0.37; 95% CI [−2.97; 3.72] mg/dL, *p* = 0.82) and HDL-C (0.52; 95% CI [−0.71; 0.59] mg/dL, *p* = 0.85) between IF and no-intervention groups.	-

Key: ↓ decrease; ↑ increase; - not studied. IF = intermittent fasting; CR = continuous restriction; CER = continuous energy restriction; TRE = early time-restricted eating (skipping dinner); BW = body weight; BF = body fat; BMI = body mass index; SBP = systolic blood pressure; DBP = diastolic blood pressure; TG = triglycerides; LDL-C = low-density lipoprotein cholesterol; HDL-C = high-density lipoprotein cholesterol; WMD = weighted mean difference; TC = total cholesterol; CI = confidence interval.

## Data Availability

No new data were created or analyzed in this study. Data sharing is not applicable to this article.
